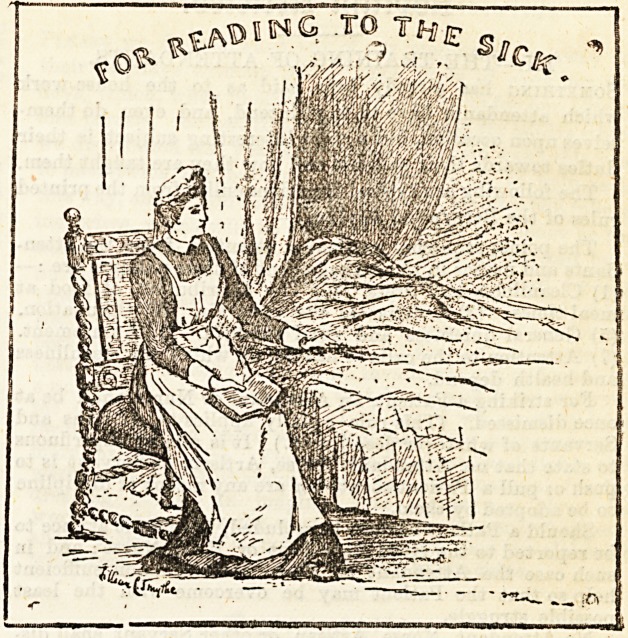# The Hospital Nursing Supplement

**Published:** 1891-07-18

**Authors:** 


### "The Hospital" Nursing Mirror.

**Being the Extra Nursing Supplement of "The Hospital" Newspaper.**

Contributions for this Supplement should be addressed to the Editor, The Hospital, 140, Strand, London, W.C., and should have the word "Nursing" plainly written in left-hand top corner of the envelope.

### En Passant.

**Examinations at the London.**—The probationers of the London Hospital have done particularly well at their annual examination this year; 73 went up and 69 passed. The first prize has been gained by Probationer Beale, while the second falls to Probationer Baillie, and the third is divided between Probationers Blackett and Irving. Honorary certificates have been awarded to Probationers Townsend, Steen, and Winter; the certificates are signed by Dr. Anderson and Mr. Mansell Moullin. We hope to give the examination questions next week.

**Our Friends Abroad.**—We print this week a short but very interesting letter from Dr. T. Duncan Greenlees, who two years ago allowed us to publish in these pages his introductory lecture to the nurses of the City of London Lunatic Asylum. It is pleasant to find that Dr. Duncan Greenlees is still one of our readers, though his professional work has taken him so far afield. A friend tells us that having advertised in our pages for a nursery governess, she got several answers from abroad, as well as the usual rush from women in England. All this is very satisfactory, for our great desire is to get into touch with nurses all the world over, to find out where there is progress, to gather news and hints.

**A New Zealand Pro.**—Somehow New Zealand seems somewhat out of reach of our correspondents, and now we have a letter from there it is not altogether satisfactory. There is a London ring about it—it is the grumble of a pro.! However, our colonial pro. did not grumble about tinned lobster; she was a wee bit wider minded than that; she took up the cudgels on behalf of two patients whom she alleged were neglected at the Auckland Hospital. Both patients are dead, and there is no one to support the statements of Miss Arnaboldi, and the Committee have snubbed and dismissed the unfortunate probationer in a manner we cannot but regret. The Board curtly rejected the following proposal from Mr. Lennox: "That the reports of the Superintendent of the Hospital, and Drs. Philson and Hooper, be referred back to the House Committee, with power to examine the nurses of whom Probationer Arnaboldi spcke." This is a pity; every charge should be fully enquired into, even when it is only an ignorant pro. who stands up to judge a medical superintendent old enough to be her father.

**Royal National Pension Fund.**—On Saturday last a conference of hospital workers on the subject of this fund was held at Birmingham, the Mayor presiding. The Rev. J. C. Blissard, Miss F. Shelton, and many nurses were present. Mr. Henry C. Burdett addressed the meeting, and explained the objects of the fund. He reminded the younger nurses that if they had thoughts of marrying they could join one of the returnable scales and withdraw their payments to secure their trousseau, but he advised all to oin young, as the payments were then so much smaller. They had on the Committee of the fund Lord Rothschild and some of the most eminent financiers of the day, who gave their services gratuitously, so that there was every guarantee that the fund would be well managed and soundly and profitably invested. In two years they had accumulated and invested £100,000; the donation and bonus fund amounted to £40,000, and the benevolent fund to £10,000. To those who had saved money already the fund offered greater advantages than the Post Office. For one thing, money paid in on the returnable scale could be willed away. The usual votes of thanks concluded the proceedings.

**Quiet Nooks.**—The Great Eastern Railway issues a pamphlet called "Quiet Nooks," which gives particulars of Overstrand, Trimington-on-Sea, and other places on the North-East Norfolk coast where lodgings are to be had. It also issues a pamphlet on "Farmhouse and Country Lodgings," giving a list of houses in various villages in Essex, Herts, and Suffolk where lodgings can be had during the summer months. Possibly these pamphlets might be useful to nurses who have not yet made their holiday arrangements; if so, they can be had on application to the Superintendent of the Line, Liverpool Street Station, London, E.C.

**Midwives Again.**—At the Central Criminal Court on July 8th Mary Griffiths and Mary Walters, middle-aged women, who acted as midwives but were not members of the L.O.S., were charged with causing the death of Lucy Edmunds, a married woman, by their negligent treatment. Griffiths was acquitted, but Walters was sentenced to three months' hard labour. The whole aspect and conduct of the women was suitable to charwomen, but not to those who profess to be skilled practitioners. There was an interesting article in last week's *British Medical Journal* on "Midwives in Austria-Hungary"; we advise those of our readers who are midwives to peruse it.

**Short Items.**—Mrs. Grimwood went to Windsor on July 2nd, and was presented to the Queen, who bestowed on her the decoration of the Royal Red Cross.—An anonymous donor has given £200 to finish the furnishing of the Home of Rest for Nurses.—Miss K. Maguire headed the list at the late medical examinations in connection with the Adelaide Hospital, Dublin.—The *British Medical Journal* gives the following answer to a physician which will interest monthly nurses: "An engagement made for a definite date and not broken by the nurse must be held as entitling her to compensation."—Sister Mary Baptist, Matron of St. Mary's Hospital, San Francisco, is the sister of Sir Charles Russell. —Sister Margaret, during a fire at an asylum for deaf mutes, saved 140 of the inmates by making a rope of sheets, and lowering them from the windows.—Miss Bates is now matron at Steyning; Miss Stockwell is private nursing for the Sussex County Hospital.

**Doctor and Nurse.**—An Irish paper has commenced a series of articles on nursing by a physician who is much given to the use of italics, but who writes forcibly on subjects of common foibles. This physician seems to have come across many nurses (probably they were amateurs) who had not been trained to obedience, therefore he says let the nurse reflect "that it is *not diseases* altogether *but patients* who are treated; that cases of the same disease require opposite treatment, according to the state of the man attacked, and that what she has seen do great good in another case of the same disease, and apparently similar in every way, might kill this patient. Besides, it is the doctor who is responsible for the line of treatment adopted, not the nurse. Let her discharge her own duties efficiently, and she will find enough to fully employ the most gifted woman, and allow the doctor a fair chance for his line of treatment." Again he says: "There is one great popular fallacy which is shared by most nurses with regard to doctors and medicine, which is, quite unjustly, the cause of want of confidence and of failure to exactly follow prescribed directions. It is the belief in specifics. Most people fancy there is a certain remedy which is best for each disease, or at least a certain line of treatment, and they consider that where doctors usually fail is in hitting on the proper remedy or remedies. The fact is, there are *very few, if any, such things as specifics.* Perhaps the nearest approach to a specific is the action of quinine in curing ague or intermittent fever. But though the cure is almost certain in the usual run of cases, yet *many circumstances in the patient's condition* might render the use of quinine almost a crime, and the doctor would have to resort to something else."

### Lectures on Surgical Ward Work and Nursing.

By Alexander Miles, M.B. (Edin.), C.M., F.R.C.S.E.

### Lecture XXX.—BANDAGES FOR THE GROIN AND PERINÆUM.

Bandages for the Groin are used to retain any form of surgical dressing or apparatus in position, and also in the treatment of hernia. The spica is the form of bandage selected in this region, and it may either exert pressure from below upwards or from above downwards, according as the ascending or descending spica is applied. The ascending spica, in which each succeeding turn goes higher up the limb than its predecessor is that usually chosen when fixing a splint or a dressing. In exerting pressure on a hernia, however, you vary the form of spica according as you have to deal with a femoral or an inguinal hernia. Thus, you remember that an inguinal hernia leaves the abdomen at the internal abdominal ring, passes *downwards* through the inguinal canal, out through the external ring, and continues to pass *downwards* into the scrotum. Therefore, to return this form of hernia to the abdominal cavity, and to retain it there, pressure must be exerted *upwards*, and this is effected by the ascending spica. On the other hand, in femoral hernia, although in the first instance the bowel passes downwards in the crural canal, when it emerges from the saphenous opening, its direction is changed, and it passes *upwards* over the front of the abdomen. Hence, for its reduction pressure must first be from above *downwards*, the direction in which a descending spica presses.

Ascending Spica of Groin.
First turns. Others follow at slightly higher level.

(*a*) *Ascending Spica of Groin.*—Place the tail of the bandage over the external abdominal ring, that is, at the upper and inner aspect of the groin, on the ruptured side, and thence carry a turn round the pelvis, going towards the *same* side, back to the point from which you started. This is one loop of the figure-of-eight. The other is made by continuing the bandage across the front of the thigh, round its outer and posterior aspects and into the perineum from behind, again reaching the starting point. Each turn overlaps two-thirds of the one before it, and with three or four such turns the bandage is applied. Note that you go round the *pelvis*, not the waist, which is movable, and so permits the bandage to become loose.

(*b*) *Descending Spica of Groin.*—Again begin with the tail over the point from which the hernia emerges, in this case the saphenous opening. Then carry it round the pelvis, going first towards the *opposite* side, however, and so it will come across the front of the thigh and enter the perinæum from the *front*, thence round the outside of the limb, back to the starting point. The following turns pass from above downwards, and exert their pressure in this direction.

(*c*). *Double Spica of Groin.*—This bandage may be used for fixing dressings to both groins, but in the treatment of hernia would only be applicable in cases where there was an inguinal hernia on one side, and a femoral on the other, as one side is an ascending, the other a descending spica. Such a combination of herniæ is rare, and when you have to deal with a double hernia of the same kind apply two similar bandages, rather than the double spica. To apply it make one figure-of-eight as for an ascending spica, say on the left side, and, then instead of making a second, cross the middle line of the body and make a figure-of-eight round the right thigh. This will be a descending spica. Repeat these turns alternately till both groins are covered in.

### Bandages for Perinæum.

The "St. Andrew's Cross bandage," or looped bandage, for the perinæum, is useful for retaining dressings on that part. It consists of a series of loops applied alternately round the pelvis and across the perinæum. Begin by laying the tail of the bandage over the right side of the pelvis, and make a turn round the body so as to catch in and so fix the tail. Now pass across the front of the right thigh into the perinæum and, *crossing the middle line*, let the bandage pass round the back of the left thigh and across the buttock to the pelvis, round which a turn is made, and then the second perineal turn is made in the same way as the first, only from the opposite side. The two turns cross in the centre of the perinæum, and so form a St. Andrew's Cross. Similar turns are added till the dressing is secured.

The T-shaped bandage for the perinæum is made by sewing together two pieces of bandage so as to form a letter T. The horizontal part of the letter is to encircle the pelvis, with the vertical part hanging down behind. The latter is then brought forward between the thighs, and fixed to the former, and so a perineal dressing is retained in position. By using this bandage dressings can be frequently changed without much disturbance to a recumbent patient.

Handkerchief Bandages for Lower Extremity.—The handkerchief bandage originally used by Gerdy and Mayor of Lausanne, but usually associated with the name of Esmarch of Kiel, is particularly useful for temporary and emergency dressings, and was largely used by Esmarch in military surgery. They may be square or triangular in shape, the latter being the more generally useful. The base of the right-angled triangle should be a-yard and a-half long, and the material from which it is cut should be at least one yard wide. The ends should be tied into a reef-knot or bow, or fixed with a strong safety pin.

For the Foot.—The base is folded up for a short distance *en cravatte*, and then the foot is laid on the handkerchief, the apex being well beyond the toes. The edges are neatly folded up over the foot, and then the base is passed round the ankle and instep and the ends secured.

For the Knee.—The triangular handkerchief is laid over the dressing and the ends brought round and firmly tied, the loose ends being carefully folded in.

For the Hip or Buttock.—Two triangles are required. The first is folded *en cravatte*, and tied round the pelvis as a belt. The second is held with the base downwards and the apex up, the intermediate part covering over and securing any necessary dressing. The base passes round the upper part of the thigh, and the apex is pushed between the patient's skin and the belt, and then folded down and secured with a safety pin.

There are many other applications of this form of bandage figured by Esmarch in his "Surgeons' Handbook."

### Nursing Medals and Certificates.

ST. JOHN THE EVANGELIST.

ST. JOHN'S HOUSE was founded in 1848, and had two ranks of Sisters; the Grey Sisters, who were required to work for six months in the year, and the Blue Sisters, who were supposed to belong entirely to the sisterhood. Both ranks wore the above silver cross, but the Blue Sisters had an eagle on their cross. The nurses wore a medal, on a narrow piece of blue ribbon their first year, on a broad piece subsequently. All Sisters and nurses were admitted at a religious service when the cross or medal was slung round their necks. When St. John's House was finally given up, those who belonged to it retained their badges as a remembrance. The Sisters of St. John the Divine wear the above cross now.

### The Second Thousand.

H.R.H. THE PRINCESS OF WALES will receive the second thousand members of the Royal National Pension Fund at Marlborough House on Saturday, July 25th, at noon. Either indoor or outdoor uniform may be worn. All nurses who wish to be present at Marlborough House are requested to be present at Merchant Taylors' Hall, on Thursday night, 23rd inst., when they will be taught in what order to approach the Princess. All members of the Pension Fund who write to the Secretary and send a stamped addressed envelope, can have an invitation for Thursday night, and we expect a large number will avail themselves of this offer, for a delightful entertainment has been arranged. The Countess of Cadogan will receive the nurses; Mr. Harry Furniss will give his amusing Parliamentary sketch; the band of the Royal Engineers will be in attendance; Messrs. Ring and Brymer will again supply the refreshments, and all augurs well for a pleasant conversazione and the meeting together of many old friends. We hope all nurses will come prepared to enjoy themselves. Mr. Burdett will be glad to hear from any member of the first thousand who was unable to attend the Marlborough House reception last year, but who could do so this year; when every effort will be made to secure invitations for these nurses. The railway companies have generously agreed to issue return tickets at a single fare to all nurses attending the conversazione and reception by the Princess, on producing their cards of invitation to the booking office clerks.

COURAGE.

"Cowards die many times before their deaths,
The valiant never taste of death but once."

THE discomforts of fear are are so obvious that we will look at some of the advantages of being brave. It is impossible to value them too highly since courage gives calmness and confidence in danger, and we shall be wise if we strive our utmost to gain it. Some persons are constitutionally braver than others; with good health and strong nerve they dare and do where a timid soul in a weakly body, with equally good intentions, shrinks back and dares not. Others have an insensibility to danger which makes them live carelessly at the brink of a volcano, quiet and secure like the Sidonians of old till their destruction overtakes them suddenly. They are no more to be commended than the coward who sees ever a "lion in his path" and stimulates himself with "Dutch courage" to meet it, but finds still greater timidity and depression when the effect has passed away.

True courage is built on the sure foundation of faith and trust in a God who never fails nor forsakes those who love Him. If He is for us what matters it who is against us?

We all feel our hearts glow and burn within us and the ready tear start at the memory of noble deeds. The fireman braving the flames to save the weak from a fearful death, the signalman giving his own life to prevent a train being wrecked, Sir Philip Sydney at Zutphen refusing the cup of cold water at his dying hour that another might have the refreshment he so craved for, and later General Gordon in Africa, and Lieut. Grant at Manipur. Such glorious actions are happily not rare, and we rejoice that such men are of our own kith and kin. We must not imagine that these men became heroes all in a moment. Of what we can gather of their early lives they had simply done their duty wherever they found it—had been patient, unselfish, God-fearing for years, so that when the hour of trial arrived they blossomed out into heroes as much to their own surprise as to that of other people.

Few of us are called on to make supreme efforts for our fellow creatures, but we can all bear pain patiently if we try, or take a loathsome draught in a courageous spirit. A house companion who is continually "nagging" and opposing our wishes and little pleasures, or who scorns alike our weaknesses and our highest aims, may make heroic stuff of us if we bear patiently and in the spirit of our great hero, Christ. Study His character to gain a lesson of forbearance, of helpfulness, of love to our enemies, of a life laid down. Imitate Him and our lives will flow on like the mighty river which is,

"Tho' deep yet clear, tho' gentle yet not dull,
Strong without rage, without o'erflowing full."

### Asylum Articles.

III.—THE TRAINING OF ATTENDANTS.

Something has already been said as to the house-work which attendants have to superintend, and even do themselves upon occasion, but a more interesting subject is their duties towards their patients and how they are taught them.

The following are typical directions culled from the printed rules of the Berrywood Asylum :—

The points requiring particular attention from the attendants and nurses in the management of their patients are :—(1) Cleanliness and dress. (2) The distribution of food at meal times. (3) Exercise in the open air. (4) Occupation. (5) General quietness and good conduct. (6) Amusement. (7) Attention to the calls of nature, on which all cleanliness and health depend.

For striking a Patient, an Attendant or Nurse shall be at once dismissed. (This rule equally applies to Artisans and Servants of whatever description.) It is almost superfluous to state that no Attendant, Nurse, Artisan, or Servant is to push or pull a Patient about, nor are any means of discipline to be adopted by them.

Should a Patient have to be secluded, the fact is at once to be reported to the Head Attendant or Head Nurse: and in such case the Attendant or Nurse shall summon sufficient help so that the Patient may be overcome with the least possible struggle.

No Attendant, Nurse, Artisan, or other Servant shall discuss the affairs of the Asylum outside of it ; they shall not mention the names of Patients ; and they shall refer anyone who asks for information as to the state of any Patient to the Superintendant.

Any accident or illness, however slight, must be at once reported to the Head Attendant or Head Nurse, who will immediately see the Patient, and, if necessary, inform the Medical Officer. In the same way must be reported any scuffle or struggle an Attendant or Nurse has had with any Patient, whether or not it shall have resulted in any apparent injury to the Patient.

An epileptic Patient seized with a fit must be placed as soon as possible on a bed or sofa, and have the clothing about the neck and chest made loose and free. A succession of fits causing prostration should be reported without delay, as well as any fit occurring in any patient who had not been epileptic before.

As for other regulations about the use of safety matches, the prevention of patients from eating berries, &c., when they are out walking, the careful search [for contraband or dangerous articles hidden in the bedding, they are all hints which train the junior nurse to understand on what a narrow path she is treading. The least slip or forgetfulness and dire may be the results.

The theoretical training of the attendants is at present a burning subject, and for any suggestions offered here the writer is indebted to Dr. Harding and Miss Evans, of Berrywood, who (in the absence of Dr. Greene) were ever ready to answer questions or talk on the [subject which both have at heart. Dr. Harding was of opinion that any hospital-trained nurse who would enter an asylum at the bottom of the ladder and be content to serve exactly as the other attendants, was sure of rapid promotion, and if she stuck to the work and was suited for it, was sure to finally obtain a post as Matron. On the other hand, Miss Evans had tried in the wards at Berrywood two trained nurses, and both had proved failures ; one had to be dismissed for striking a patient

The present system at Berrywood is to train all the attendants in the same way that nurses are trained, namely, by daily experience and by lectures. The Medical Officer and the Matron in going their rounds "point a moral" to the younger nurses whenever possible ; during the winter months lectures are given, the first course was the First Aid of the St. John Ambulance Association, the second course will be on nursing, pure and simple. Dr. Harding's view of the certificates of the Medico-Psychological Association was in amusing confirmation of the views of nurses with regard to outside certificates, that in so for as they dealt and could deal, only with theoretical knowledge, they could never be of much practical value. The natural answer to this, however, is why should not asylums give certificates or badges, to those nurses who serve them faithfully for three years? Why should our list of "Nursing Medals and Certificates," not contain a shield or cross, with proper inscription, duly given to every trained attendant? The hospitals have first claim on the cross by right of priority, so we recommend asylums to adopt the shield.

### Cape Colony as a Field of Labour for English Nurses.

T. Duncan Greenlees writes : The English market seems to be now well-nigh overstocked with skilled labour, and as a result the market value has correspondingly deteriorated. In view of this fact, might I point out to your readers, especially asylum nurses, several of the advantages of this Colony when viewed as a field for skilled labour? In the first place, the pay is much greater than at home. In my own institution the salary begins at £40 per annum, and this is increased to £50, after a year's satisfactory service. In addition, board, lodgings, and uniform are provided, so that, although the cost of living may be slightly higher than it is at home, yet the dear articles are mainly to be found in one or other of the above three items, and a girl could easily save from £30 to £35 per annum, if so inclined. Secondly, the climate is delightful. It is now midwinter, and, although chilly at night, yet during the day the temperature is that of an English July, and snow or ice are rarely seen. In the summer-time it is hot, but never so warm as to make life a burden as is often the case in India. The steppes of Africa are among the most healthy spots in the world for cases of weak chest, the air being dry and bracing. Thirdly, the voyage out is a most pleasant experience—three weeks of a lazy, happy life, exposed to the ozone-impregnated air is just sufficient to give the exhausted mind and body a healthy fillip. Further, flying visits to Lisbon, Madiera, &c., are full of pleasurable excitement. Lastly, life in the Colony differs little from that in England. True, the advantages—and how few they are!—of modern civilisation, as met with in London, are absent, but their place is well filled by the kindly feeling every colonist bears to one another, and the distance separating us from our friends at home is but a tie to bind us closer together.

Grahamstown, June 15th, 1891.

### The Princess of Wales' Fund for Mrs. Grimwood.

Miss Knollys acknowledges the following sums received towards the Princess of Wales' Fund for Mrs. Grimwood: From a Matron, 5s.; Nurse Byrne, 3s.; Nurses Proschidetzky, E. M. Leake, R. Hammond, S. A. Steer, E. C., and Mary Ann Hay, 2s. each; Nurse Alice Campbell, 5s.; Nurse M. Smith, 5s.; Nurses Ellen Chapman and C. M. Martin, 2s. 6d. each; Nurses E. Parker, M. Parker, Alloway, J. R. D., "W.," M. E. Spreat, A. L. Spreat, C. Dunn, Alice, Phillis Legate, L. A. Smith, F. Blann, Sarah Ann, Emma, Jane Ellen, Charlotte, Jane, Edith, Ada, Janet, Mary, Ada Frances, Pauline, Pollie, Annie, Fanny, M. A. Payne, Elizabeth Burton, A. Lee, Barbara, Penelope, M. Smith, and Lowe, Sisters Ella, Ada, and Maude, all 1s. each. We remind other members of the First and Second Thousand that if they desire to show their appreciation of the kindness the Princess of Wales is showing them, they cannot do better than send a P.O. for a shilling to Mrs. Grimwood's Fund, care of Miss Knollys, Marlborough House, Pall Mall, London.

### Everybody's Opinion.

*[Correspondence on all subjects is invited, but we cannot in any way be responsible for the opinions expressed by our correspondents. No communications can be entertained if the name and address of the correspondent is not given, or unless one side of the paper only be written on.]*

### DISTRICT NURSING.

"AN OLD DISTRICT NURSE" writes: While agreeing with much of the letter of "E. H." in last week's HOSPITAL, will you kindly give me space for a few remarks? District nursing in many small towns and country villages is quite a new undertaking. The mistake made by those is often in the selection of a nurse. Perhaps one is engaged who is an excellent hospital nurse, but totally unaccustomed to visiting the poor in their own homes. Now, in a case like this, a local untrained "Superintendent" or lady visitor may, if a sensible person, be of very great use to the inexperienced district nurse by giving her information about the ways of the poor, &c., and also by supplying nursing appliances and linen. If the lady is wise, she will leave all strictly nursing details to the nurse—of course, acting under the doctor. This is the favourable side of the question. I am sorry to say, however, that the state of affairs "E. H." complains of is by no means uncommon. If the nurse is really well trained, and knows her work, the interference of the untrained "Superintendent" will, naturally, be most irritating. If, on the other hand, the nurse is neither well-trained nor conscientious (there are such beings), the quality of the work invariably suffers. I speak from my own experience. The "Superintendent" allows and encourages the nurse to take on more cases than she can reasonably attend to. Not having time to give to all, the standard of the nursing does not keep up. I think the remedy for this state of things (I am sorry to say, not uncommon) would be, when engaging a district nurse, always to inquire if she has been trained in district as well as hospital nursing, two distinct branches of our profession, as many of us know. Of course, in a district nursing society, where there is more than one nurse, a really well-trained Superintendent is essential to the efficiency of the work. Still I know of more than one society where the nurse, after one year of hospital training, was launched into a district without any trained supervision. The work, of course, deteriorates very much, as the nurse never comes in contact with one who is a better nurse than herself, and never likely learnt anything new, nor how to improve on the old. Her so-called "Superintendent" is satisfied with her work, not knowing any better. This is, of course, commoner in country districts and small towns. I should hope there are very few, if any, large towns where there is not a good staff of nurses, worked well and heartily, under a trained Superintendent.

### Notes and Queries.

TO CORRESPONDENTS.—1. Questions or answers may be written on post-cards. 2. Advertisements in disguise are inadmissible. 3. In answering a query please quote the number. 4. A private answer can only be sent in urgent cases, and then a stamped addressed envelope must be enclosed. 5. Every communication must be accompanied by the writer's full name and address, not necessarily for publication. 6. Correspondents are requested to help their fellow nurses by answering such queries as they can.

### Queries.

(20) Will any nurse kindly sell or tell me where I can get a good manual on midwifery, with illustrations?—*Overton.*

(21) Can any nurse give me the address of cheap, comfortable apartments at Ramsgate, Margate, or Yarmouth, where I could take an invalid patient for a fortnight?—*C. Reader.*

(22) Where can I get Dr. Allan's "Notes on Fever Nursing"?—*Nurse B.*

### Answers.

*Superintendent.*—In THE HOSPITAL for February 27th you will find the forms of certificates given at Liverpool, and on them you can found the wording for your own certificate.

*Caps.*—We have had so many queries about cap patterns that we have arranged to give some illustrations of the chief uniform caps shortly.

*An Old St. John's Nurse.*—Gunter Grove, Lewisham, Poplar, St. Gabriel's for Infants, Littlehampton Home, St. John's Hospital for Women, Drayton Gardens, District Home. Possibly you thought your query was sarcastic. See THE HOSPITAL for March 16th, 1889, or see the last report of St. John the Divine.

### Christmas Competitions.

PLEASE we do not want any of our readers to go away for their holidays without taking some piece of work for wet days, which, when finished, can be sent to us for our Christmas parcels. So heartily were the garments for adults which we distributed last year appreciated, that we want this year to have twice the number. To encourage all to help us in this way, and to add interest to the work, we offer the following prizes, which will be awarded in books or money as the winners choose: (1) For the best pair of socks knitted by a nurse, 5s.; (2) for the best pair of socks knitted by any HOSPITAL reader, 5s.; (3) for the best made flannel shirt, 10s.; (4) for the best made woman's blouse, 10s.; (5) for the best made flannel petticoat, 10s.; (6) for the best made and best shaped dressing gown for an invalid cut out and made by a nurse, 20s. It will be seen that No. 1 and 6 are reserved for nurses only. With regard to No. 6 we specially hope for many entries, and if we secure them, we propose to give more than one prize. Flannellette is cheap, and light, and warm, and would therefore form the best material for the dressing gown. In judging, four marks are given for workmanship, four for shape, and two for general appearance; therefore, it is not wise to spend time on elaborate trimmings. Long seams may be done by machine.

### Presentation.

ROYAL CHEST HOSPITAL, CITY ROAD.—Miss Mary Leslie Smith, on resigning the post of Matron at the Royal Hospital for Diseases of the Chest, City Road, after fifteen years' service, has been presented by the past and present resident medical officers with a handsome marble and bronze timepiece, with suitable inscription, together with a pair of bronze candelabra, as a token of their esteem and regard. The Sisters have presented her with a gold brooch, and the nurses with a silver scent-bottle.

### Wants and Workers.

SISTER KATHERINE, St. Mary's Nurses' Home, Plaistow, E., would be glad of the copies of THE HOSPITAL from August, 1890, to March, 1891, offered by K. W.

*The Melicent Home.*—The Matron writes that her appeal of last week has brought an offer of help from one worker, but that she would be glad to hear from others.

*Help Wanted.*—Sister Hilda, The Nursing Home, Witney, Oxford, writes that she needs outside help badly. The Home is now being removed into larger premises, and this removal will cost £50. Any donation, however small, towards this sum, will be received most thankfully. May we suggest that any readers in the neighbourhood might go and inspect the Home and its work, and then do what they can to help? The Home is for invalid children.

### Amusements and Relaxation.

### SPECIAL NOTICE TO CORRESPONDENTS.

**Third Quarterly Word Competition commenced July 4th, 1891, ends September 26th, 1891.**

Competitors can enter for *all* quarterly competitions, but no competitor can take more than *one first prize* or *two* prizes of any kind during the year.

The word for dissection for this, the THIRD week of the quarter, being

"BANQUET."

| Names. | July 9th. | Totals. | Names. | July 9th. | Totals. |
|---|---|---|---|---|---|
| Paignton | 49 | 49 | Nurse Ellen | 44 | 44 |
| Psyche | 48 | 48 | Christie | 44 | 44 |
| Hope | 47 | 47 | Dulcamara | 44 | 44 |
| Lightowlers | 47 | 47 | Nurse J. S. | 43 | 43 |
| Wizard | 47 | 47 | Qu'appelle | 42 | 42 |
| Wyameris | 46 | 46 | E. M. S. | 40 | 40 |
| Dove | 46 | 46 | Jenny Wren | 38 | 38 |
| Punch | 46 | 46 | Carpendium | 37 | 37 |
| Ivanhoe | 45 | 45 | Grannie | 36 | 36 |
| Tinie | 45 | 45 | Nurse G. P. | 35 | 35 |
| Agamemnon | 45 | 45 | Goodnight | 24 | 24 |

N.B.—Each paper must be signed by the author with his or her real name and address. A *nom de plume* may be added if the writer does not desire to be referred to by us by his real name. In the case of all prize-winners, however, the real name and address will be published.

All letters referring to this page which do not arrive at 140, Strand, London, W.C., by the *first post on Thursdays*, and are not addressed PRIZE EDITOR, will in future be disqualified and disregarded

### In a Devonshire Village.

(*Concluded from page* lxxxviii.)

I KEPT close watch in the sick room all day. Towards evening Mrs. Gray came, as she always did, to take my place for a few minutes while I went out to get some fresh air. On one of my patient's "bad days" I sometimes did not go further than round the farm. This evening, however, I turned my steps towards the further end of the village, where, at the end of a short shady lane, stood the small church.

The bell was ringing for evensong, but very few of the villagers (most of whom were at work in the fields) attended this daily service.

I met no one on the way except a young man, somewhat travel-stained, who, seated on the bank in the lane, consulting a guide book, was evidently doing a walking tour, and looking up the neighbourhood.

He glanced up as I passed, and the thought struck me that I had seen his face before, but I could not recall where.

I hurried on to the vestry, and handed my request to the clergyman as he was about to enter the church.

I found the congregation consisted of some old people from a neighbouring almshouse, a few school children, and myself.

The service had hardly begun when the stranger from the lane entered, and sat down in one of the free seats near the door. He carried a fragrant bunch of woodruff in his hand, and the scent of that most delicate flower is for ever associated in my mind with the quiet service of that summer evening.

I did not see the stranger's face again. He knelt with it deeply buried in his hands, while my thoughts flew back to the small sick room I had just left, where the weary struggle for life was still going on.

Could it be that the struggle was now drawing to a close, that soon the summons would come to the soldier to leave his post where he had been so long "on duty"?

My thoughts were still with him as we sang—while the evening sun fell through the western window and cast a mellow light into the little church—

> "The golden evening brightens in the west,
> Soon, soon to faithful warriors comes their rest,
> Sweet is the calm of Paradise the blest."

At the close, before repeating the final prayers, the clergy man turned and said :—

"The prayers of the congregation are desired for Robert Stewart Newman, who is seriously ill."

With a sudden start the stranger raised his head and gazed at the clergyman as if he did not trust his ears.

He half rose from his seat, sat down again with a face white and set (the likeness to someone I had known seemed more striking than before), then, burying his face in his hands, he remained motionless until the end of the service.

I hurried out, fearing I might already have been away too long, but I had hardly reached the lane before I was overtaken, and a voice spoke huskily and quickly in my ear—

"Excuse me, I see you are a nurse. You may be able to tell me. Do you know anything of the Robert Newman who was prayed for in church? It may be only a coincidence in the name, but I must find out where he is."

"The Robert Newman who was prayed for is my patient at present," I replied as calmly as I could, for I felt excited myself, and my brain was full of wild fancies. I guessed now why his face had seemed so familiar to me.

"May I go with you? Can I see him? Is he *very* ill?" The questions came faster than I could answer them, and he impatiently changed his pace into such a quick walk that I could hardly keep up with him.

"He is seriously ill," I replied, "and must certainly not be excited. If you will come with me I will, if he seems able to bear it, convey to him any message you may wish to send him, but I cannot promise more. I cannot promise that you may see him yet."

Then question followed question, until my companion had learnt all I could tell him. How Mr. Newman had been passing through the village and had fallen ill, how lonely he was, and how good and patient during the long weeks of illness. I spoke of the books he had; of his longing to see his only son once more.

I could keep nothing back, and I had to repeat more than once what the doctor had said on his last visit, and what my own opinion was of the case, and of the chance of recovery.

We entered the gate, and crossed the farmyard together. I stopped at the porch.

"I will go up," I said, "and will let you know at once how I find him. Shall I give him your name?"

"My name," he replied, looking steadfastly at me, and speaking in a low voice, "my name is Robert Stewart Newman."

My patient was awake, and seemed a trifle better. Would the news be too much? Could I—dare I keep any longer apart these two, who should never have been separated?

Would joy undo the work that sorrow had done, and bring back once more the light of life to those weary eyes, the smile of happiness to that sad face, the wish once more to live to that sorrowing heart—to live because there was now someone to live for, or would the shock be too great? Would the feeble strength be unable to bear the rush of hopes so suddenly and unexpectedly fulfilled?

I decided in the few moments I paused to look at him. "I have been to church, Mr. Newman," I said quietly, sitting down by the bedside, and holding his hand, "and prayers were asked for your recovery."

"The prayers have been answered," I added; "the answer will make you very happy."

I paused, for I found I was speaking in enigmas to him. How should I tell him the truth?

"There was someone in church who seemed to know your name, he asked leave to see you, and he is downstairs now, waiting to know if he may come up."

My voice faltered, and I could say no more.

A sudden light of hope lit up the wasted face. I did not lose a moment, but ran downstairs. "Go quietly," I said, "do not startle him, I think he guesses who is coming."

When I followed him some time after, I found my place taken by the sick man's bedside. They had forgotten my existence, indeed had forgotten everything but the fact that they were now together once more.

In the quiet happy days that followed, joy did all that I longed and expected it would do, nay even more quickly than seemed possible strength came back, and it was not long before I found my services were no longer needed.

"So I went my way," as Bunyan says, "and saw them no more."

I heard nothing more of my patient. This is only a short chapter in the history of two lives, of which I saw neither the beginning nor the end, but when I look back and think of those two, so long strangers to each other, and so wonderfully brought together, I feel glad at heart to know that to him who had been given "patience under his sufferings," was now added the crowning mercy of "a happy issue out of all his afflictions."

## Figures and Tables

**Figure f1:**
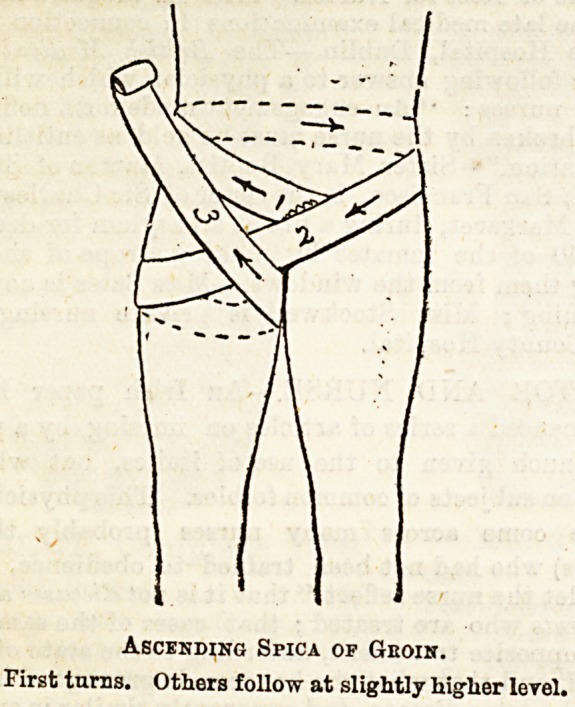


**Figure f2:**
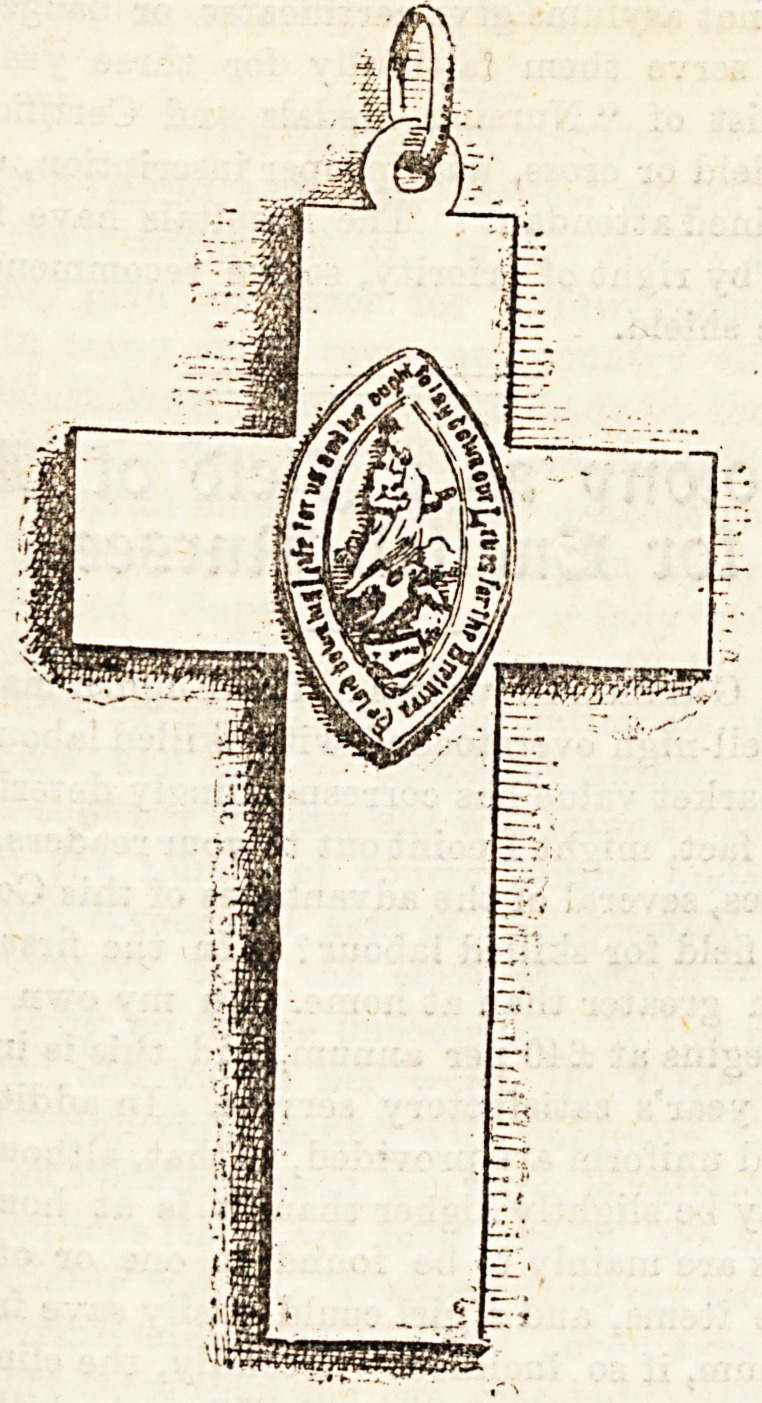


**Figure f3:**